# Muscle network topology analysis for the classification of chronic neck pain based on EMG biomarkers extracted during walking

**DOI:** 10.1371/journal.pone.0252657

**Published:** 2021-06-21

**Authors:** David Jiménez-Grande, S. Farokh Atashzar, Eduardo Martinez-Valdes, Deborah Falla

**Affiliations:** 1 Centre of Precision Rehabilitation for Spinal Pain, School of Sport, Exercise and Rehabilitation Sciences, College of Life and Environmental Sciences, University of Birmingham, Edgbaston, Birmingham, United Kingdom; 2 Electrical & Computer Engineering as well as Mechanical & Aerospace Engineering, New York University, New York City, New York, United States of America; University of Illinois at Urbana-Champaign, UNITED STATES

## Abstract

Neuromuscular impairments are frequently observed in patients with chronic neck pain (CNP). This study uniquely investigates whether changes in neck muscle synergies detected during gait are sensitive enough to differentiate between people with and without CNP. Surface electromyography (EMG) was recorded from the sternocleidomastoid, splenius capitis, and upper trapezius muscles bilaterally from 20 asymptomatic individuals and 20 people with CNP as they performed rectilinear and curvilinear gait. Intermuscular coherence was computed to generate the functional inter-muscle connectivity network, the topology of which is quantified based on a set of graph measures. Besides the functional network, spectrotemporal analysis of each EMG was used to form the feature set. With the use of Neighbourhood Component Analysis (NCA), we identified the most significant features and muscles for the classification/differentiation task conducted using K-Nearest Neighbourhood (K-NN), Support Vector Machine (SVM), and Linear Discriminant Analysis (LDA) algorithms. The NCA algorithm selected features from muscle network topology as one of the most relevant feature sets, which further emphasize the presence of major differences in muscle network topology between people with and without CNP. Curvilinear gait achieved the best classification performance through NCA-SVM based on only 16 features (accuracy: 85.00%, specificity: 81.81%, and sensitivity: 88.88%). Intermuscular muscle networks can be considered as a new sensitive tool for the classification of people with CNP. These findings further our understanding of how fundamental muscle networks are altered in people with CNP.

## Introduction

Neck pain is one of the most frequent musculoskeletal disorders, affecting more than 70% of the adult population [1, 2]. Although the clinical presentation of people with chronic neck pain (CNP) is varied, a common observation is the presence of neuromuscular adaptations. Studies using electromyography (EMG) have revealed altered behaviour of the neck muscles (e.g., increase co-activation, reduced specificity of activity, delayed onset) in patients with CNP compared to asymptomatic individuals [3–5]. Very few studies, however, have examined multi-muscle coordination and whether this is modified in people with neck pain.

It is well known that the central nervous system (CNS) performs functional tasks such as walking or running via shared functional connections [6] that can be analysed through the intermuscular coherence of paired EMG signals [7]. Muscle synergies are commonly assessed during rectilinear gait in different conditions (i.e., treadmill walking, normal gait) [8–10], but interestingly, a fine-tuning of muscle synergies was observed during curvilinear trajectories, which was considered to be a new biomechanical strategy to face the more challenging task which demands more computational cost for the CNS [6]. Considering the need for head rotation [11, 12], neck muscles present specific synergies during curvilinear gait [6, 13].

In the current work, we analyzed the coherence between different muscle groups to detect any changes in their synergistic behavior. We investigated whether it is possible to classify people with CNP from asymptomatic individuals based on EMG signals obtained from their neck muscles during rectilinear and curvilinear gait. For this purpose, a set of frequency-domain and time-domain features were extracted from the EMG recorded from neck muscles. Among the extracted frequency-domain features, we considered intermuscular coherence between groups of neck muscles. Many studies have shown its efficacy in the detection of auditory, somatosensory, and visual evoked potentials [13–15], as well as in muscle synergies at different frequency ranges [16]. Intermuscular coherence can quantify the level of functional synchronization between different muscles. The detected synergy can also be mapped into an anatomical and structural muscle network. Functional muscle networks provide graph-based information about how muscles function synergistically, which can be used to infer potential common neural pathways and their functional neurophysiological relationship (not their physical association) [17–19].

We applied the Neighbourhood Component Analysis (NCA) algorithm [20], which provides relevant information about the discriminative power of the feature space. Previous studies have applied NCA to EMG [21] and electroencephalography [22] signals, improving their classification results (for different applications) and achieving better results than Principal Component Analysis (PCA).

Different classification approaches have been applied to classify participants based on EMG measures. The most commonly used methods are neural networks, fuzzy systems, and support vector machines (SVM) [23]. Other methods such as k-nearest-neighbours (K-NN) [24] or linear discriminant analysis (LDA) have also led to excellent classification particularly when classifying different motor tasks such as hand gestures (see [24, 25], and reference therein). In this study, we compared three different classifiers, LDA, K-NN, and SVM, to determine whether supervised machine learning algorithms can be used to classify people with CNP based on EMG data acquired from neck muscles during rectilinear and/or curvilinear gait. We hypothesized that the connectivity between different muscle groups will be modified in people with CNP and that this measure will play an important role in the classification of people with and without CNP based on EMG acquired from their neck muscles during gait. Verification of this hypothesis would provide evidence supporting common neuromuscular adaptations in people with CNP.

## Materials and methods

### Participants and protocol

Twenty asymptomatic individuals and 20 people with CNP were recruited for this observational study. Asymptomatic participants were eligible if they had no history of a neck injury or neck pain in the last two years that required treatment from a health care practitioner. Participants who suffered from CNP were eligible if they fulfilled the following criteria: (1) had a history of CNP longer than three months and (2) reported their average neck pain intensity over the last four weeks as greater than 3 out of 10 on a Numerical Rating Scale (with two anchor points; 0 = no pain and 10 = worst pain imaginable) [26, 27]. Exclusion criteria for both groups were previous spinal surgery, rheumatologic condition, current or chronic respiratory condition, or an ongoing compensation claim related to an injury.

All participants signed an informed consent form before the experimental session. No information regarding the expected results was provided to avoid bias. The study was conducted within a Laboratory at the Centre of Precision Rehabilitation for Spinal Pain (CPR Spine) congruent with the Declaration of Helsinki principles. Ethics approval was obtained from the University of Birmingham Ethics Committee (CM06/03/17-1).

**Fig 1:**
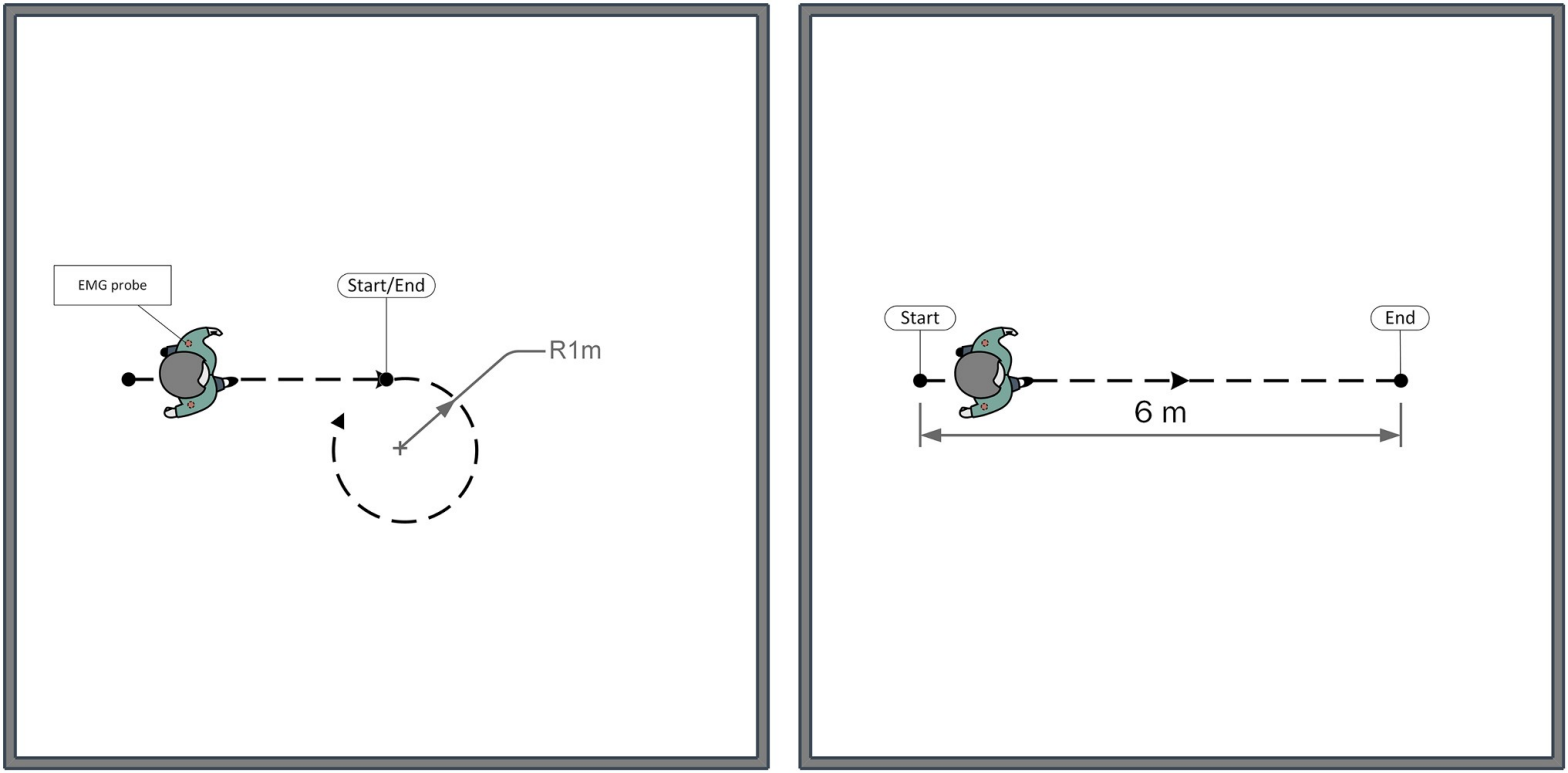
Experimental setup. Curvilinear task (left) and rectilinear task (right).

Participants were asked to walk barefoot at a self-selected speed along a curvilinear path (radius 1 meter) in both directions (clockwise and counter-clockwise) and along a rectilinear path (6 meters), three times with a 2-minute rest between trials. A trial was considered completed when the participant reached the end-point. In curvilinear gait, the start and the end-point were the same. The order of the trajectories’ performance was randomly selected by a random number generator software (see Fig 1).

### Surface electromyography acquisition

EMG was recorded from the upper region of the trapezius (UT), splenius capitis (SC), and sternocleidomastoid (SCM) bilaterally. A wireless measurement system was used to acquire the data (BTS FreeEMG, BTS S.p.A., Milan, Italy) at a sampling rate of 1000Hz with a 16-bit resolution. The skin was shaved if necessary, to remove hair and then cleaned with an alcohol swab prior to electrode placement. The bipolar surface electrodes (size: 16×12mm, BTS FreeEMG 300) were positioned following the recommendations of SENIAM with an interelectrode distance of 2cm [28]. The raw EMG signals were stored using the BTS software EMGanalyzer and then analyzed by custom scripts (Mathworks Matlab 2019b). Gait events were automatically recognized via BTS Smart Analyzer software, which identifies initial contact and toe-off by tracking two reflective markers placed on the heel and metatarsus [29].

In order to remove noise, the EMG data were processed with a zero-lag fourth-order Butterworth band-pass filter with a cut-off frequency of 10 and 500 Hz and a notch filter with a cut-off frequency of 50 Hz. The maximal EMG amplitude over an epoch of 0.5s for each muscle recording during each task was used for the normalization of the signals [30]. All signals were filtered before normalization to minimize the effects of artifacts and noise on the normalization process.

### EMG feature extraction

EMG signals can provide information about the timing and magnitude of muscle activation as well as information about the presence of shared oscillatory inputs between muscle groups (coherence analysis). In this paper we evaluate both temporal and spectral features of EMG to decode local and synergistic activities of various muscle groups. Classical time-domain features allow us to understand how muscle activity is altered when pain is present (i.e., by decreasing or increasing the level of muscle activity in the presence of pain). The functional muscle network measures allow us to understand how the synchrony between different muscle groups are affected when people have pain. The aforementioned measures are complementary and allow us to evaluate single muscle behavior and synergistic behavior between different muscle groups. In this regard, a number of well-known features were extracted from the EMG in both time and frequency domains. All features are presented in Table 1. Time features are based on statistics of the pre-filtered signal and frequency features based on the power spectral density (PSD) of the signal. Here, PSD was computed using Fast Fourier Transform [31]. Given that relatively low levels of neck muscle activity are expected during gait, a nonoverlapping window length of 500ms was selected for feature extraction.

**Table 1:** Mathematical definitions of EMG features.

Features	Mathematical definitions
Time domain	
Mean absolute value (MAV)	$MAV\;=\;\frac{1}{N}\sum_{i\;=\;1}^{N}\left|x_{i}\right|$,
	where x_i_ represents i^th^ sample of the EMG signal, and N denotes the total number of samples in a signal window.
Root mean square (RMS)	$RMS\;=\;\sqrt{\frac{\sum_{i\;=\;1}^{N}x_{i}^{2}}{N}}$
Variance (VAR)	$VAR\;=\;\frac{1}{N-1}\sum_{i\;=\;1}^{N}x_{i}^{2}$
Waveform length (WL)	$WL\;=\;\sum_{i\;=\;1}^{N-1}\left|x_{i+1}-x_{i}\right|$
Simple square integral (SSI)	$SSI\;=\;\sum_{i\;=\;1}^{N}x_{i}^{2}$
Frequency domain	
Mean Frequency (MNF)	$MNF\;=\;\frac{\sum_{j\;=\;1}^{M}f_{j}{∙P}_{j}}{\sum_{j\;=\;1}^{M}P_{j}},$
	where *f*_*j*_ represents a frequency value of the spectrum at a frequency bin *j*, *P*_*j*_ is the EMG power spectrum at a frequency bin *j*, and *M* is the length of the frequency bin.
Median frequency (MDF)	$\sum_{j\;=\;1}^{MDF}P_{j}\;=\;\sum_{j\;=\;MDF}^{M}P_{j}\;=\;\frac{1}{2}\sum_{j\;=\;1}^{M}P_{j}$
Peak Frequency (PKF)	$PKF\;=\;\text{max}\left(P_{j}\right),\;j\;=\;1,\ldots ,M.$ [25]
Mean power (MNP)	$MNP\;=\;\frac{\sum_{j\;=\;1}^{M}P_{j}}{M}$
Total power (TTP)	$TTP\;=\;\sum_{j\;=\;1}^{M}P_{j}$

#### Magnitude squared coherence (MSC) as a frequency feature

In order to assess intermuscular coherence between muscles, MSC was calculated. MSC estimates the similarity between two signals in the frequency domain, and it can be obtained by computing:

$$MSC(f)\;=\;\frac{{\left|P_{xy}(f)\right|}^{2}}{P_{xx}(f)P_{yy}(f)}$$

where *P*_*xy*_ (*j*) is the cross-power spectral density of the two time-domain signals *x*(*t*) and *y*(*t*) at a given frequency *f* and *P*_*xx*_ (*f*), *P*_*yy*_ (*f*) are the auto-power spectral densities of *x*(*t*) and *y*(*t*), respectively. Cross-power spectral density represents the cross-correlation of two different signals on the frequency domain and auto-power density the correlation of one signal with itself [32, 33]. The result of the equation is a value between 0 and 1. Zero indicates no linear relation in the frequency domain between the signals, and one indicates a perfect linear relation between them. Thus, high coherence between EMG signals indicates high functional synchrony between different underlying muscles. Coherence is, therefore a useful measure to quantify the synchrony of two EMG signals in particular when they are restricted to specific frequency ranges [34]. It was implemented per subject between the three pairs of muscles of the same side with a window length of 500ms. It should be highlighted that MSC is a central feature in this paper as it reflects the connectivity between different muscle groups and forms the muscle network [34, 35] of each individual.

### Feature selection

In order to explore the discrimination power of the features, and at the same time, improve the performance of the algorithm, the recently-developed neighbourhood component analysis (NCA) was used. NCA is a non-parametric feature selection method based on the K-NN algorithm. To select the best subset of features, NCA learns a quadratic distance metric of the input space that maximizes the performance of nearest neighbour classification. At the end, NCA provides a ranking of all the features based on their discrimination power which allow us to select a subset of the most important ones [36]. Compared to the classical reduction methods such as PCA, the NCA algorithm does not make any assumption about the distribution of the data without losing information during the reduction process. A recent study reported that NCA performs better than PCA [21]. After the implementation of the NCA algorithm, we executed three classifiers on the selected features by the NCA to compare their performance.

### EMG classification

Three different classifiers were chosen based on their performance in previous research, and to guarantee that the classification performance did not depend on the type of the algorithm: K-NN, LDA, and SVM. K-NN is one of the most used in classification tasks due to its ease of implementation as well as its speed for small datasets. In addition, K-NN has already been shown to provide very good performance in EMG classification [24, 37]. It assigns a new sample point to a class, based on the majority votes of k closest neighbours. Therefore, the number of neighbours (k) and the method to calculate the distance between points are two essential parameters for its proper performance. Here, the distance used was the Euclidian distance, and the number of neighbours was set to 5 after testing different values (3, 5 and 7). More details on the K-NN can be found in [38]. LDA is also a well-recognized method, it has a low computational cost, and it is simple to implement [39]. Unlike K-NN, LDA is a parametric method. It finds the best linear combination of features that separates the different classes assuming a parametric distribution. More details on LDA can be found in [40]. SVM is a further powerful classifier that can provide a general solution to high-dimensional problems but with a (relatively) high computational cost. SVM has been used extensively with EMG data [41]. SVM is a kernel-based classifier that can be configured with different kernel functions (lineal, polynomial or radial basis function) depending on the nature of the problem. SVM was configured with radial basic function kernel which makes it more flexible and able to handle nonlinear problems. Detailed information on the SVM classifier can be found in [42].

**Fig 2:**
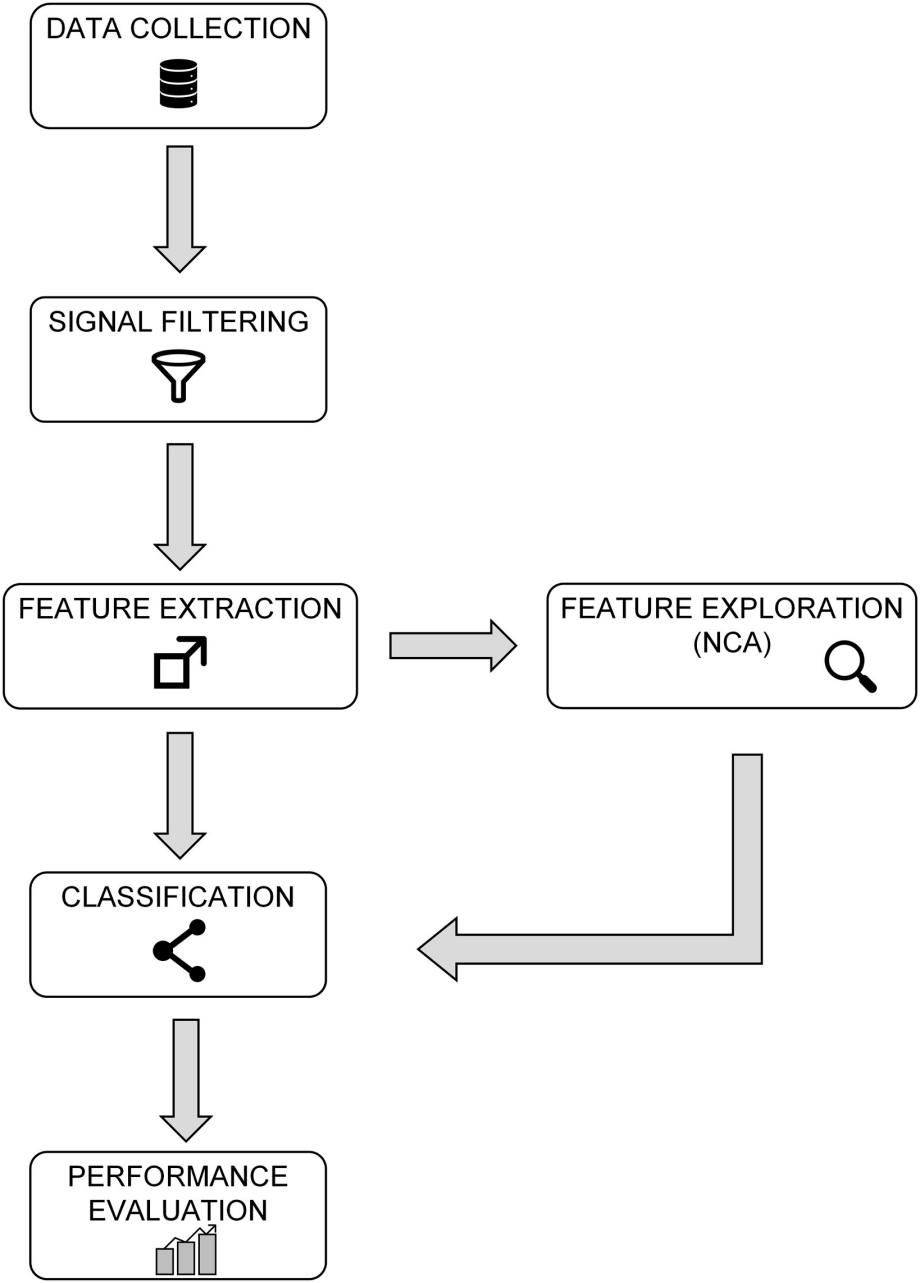
Block diagram of the methodology proposed.

In this study, the performance of K-NN, SVM (with radial basis function) and LDA are evaluated for the targeted classification problem. The complete process of the proposed methodology is shown in Fig 2.

For all classifiers, a five-fold cross-validation scheme was adopted to train their respective models. The performance of the models was analysed using the multiclass confusion matrix to obtain the corresponding values of accuracy, sensitivity, and specificity.

### Functional muscle network analysis

Intermuscular coherence can also be used to map undirected functional interactions between multiple muscles, which provides valuable information about the neural implementation of muscle synergies [43].

MSC calculation generates a value of connectivity strength for each pair of EMG signals at a specified frequency band, resulting in a weighted adjacency matrix per subject. A threshold is applied in order to emphasize only significant connections and remove those that can obscure them [33]. A proportional threshold was used to maintain the same connection density across subjects [44]. Once the average coherence matrix is obtained for each group, the connectivity between muscles can be represented graphically. In this analysis, muscles are presented as nodes, and functional connections are shown as the edges between nodes.

Muscle networks display different topologies depending on the frequency band. EMG/EMG coherence provides physiological information from both higher and lower frequency bands [45]. Indeed, significant coherence at low frequencies (<5Hz) is associated with force production, while the alpha band is related to involuntary force oscillations, called physiological tremor and the beta band, related to corticospinal projections [46]. Hence, five frequency bands of interest were considered in the calculation of the connectivity matrices to encompass the whole spectrum: delta (1–4 Hz), theta (4–8 Hz), alpha (8–12 Hz), beta (12–25 Hz), and high beta (25–30 Hz).

Therefore, we conducted a preliminary analysis using graph theory in order to explore the topology of functional connectivity between neck muscles of people with and without CNP. Comparisons of functional networks may reveal abnormalities or relevant differences between groups that can be measured by many parameters [47]. Due to the small number of muscles collected and thus the small architecture of the networks, we focused on two simple metrics, strength and betweenness centrality (BC). BC identifies hot points of high information traffic and it is calculated as the proportion of shortest paths between all node pairs in the network that pass-through a given index node. Strength is the sum of weights of links connected to the node that provides information about how strong the connections between muscles are. All measures were computed using the Brain Connectivity Toolbox [47].

### Statistical analysis

Statistical analyses were performed using custom-designed scripts in MATLAB (MathWorks, Natick, MA). Differences in age, height, weight, BMI, and muscle networks parameters between groups were conducted using Student’s t-test with a threshold of significance of 0.05. Kolmogorov-Smirnov test was used to check the normal distribution of the data. No statistical evidence was found for supporting that the data is not normally distributed.

## Results

We evaluated the performance of two different walking trajectories (rectilinear and curvilinear) with the same number and type of features in the classification of people with and without CNP through three different classifiers.

The demographic characteristics of the participants are shown in Table 2. No differences in anthropometrics and demographics were observed between groups (p > 0.05).

**Table 2:** Demographic characteristics of participants.

	Neck pain	Control	p*
	Mean ± SD	Mean ± SD	
Age (years)	28.5 ± 9.0	26.3 ± 8.8	0.33
Weight (kg)	66.2 ± 12.4	65.2 ± 13.7	0.40
Height (cm)	171.0 ± 9.9	169.1 ± 7.4	0.24
BMI (kg/m^2^)	38.5 ± 5.6	38.4 ± 6.9	0.47
Gender (female %)	60%	50%	-
Average neck pain intensity (0–10)	4.1 ± 1.9	-	-

*Independent samples t-test, SD: Standard deviation, BMI: Body Mass Index.

The overall performance of each classifier for each gait trajectory before and after the implementation of NCA is presented in Table 3. The best performance corresponds to the SVM algorithm in the curvilinear trajectory, achieving an accuracy of 85% after the implementation of NCA. In all cases, the three algorithms improved their performance after the implementation of NCA, and also, all showed better classification accuracy, specificity, and sensitivity during curvilinear gait.

**Table 3:** Classification performance for each trajectory.

		Curvilinear	Rectilinear	Combine
All features				
K-NN	ACCU	62.00%	35.00%	37.00%
	SPEC	61.90%	35.00%	37.50%
	SENS	63.15%	35.00%	37.50%
SVM	ACCU	45.00%	32.50%	56.25%
	SPEC	45.00%	31.57%	56.41%
	SENS	45.00%	33.33%	56.10%
LDA	ACCU	65.00%	42.50%	47.50%
	SPEC	65.00%	42.85%	47.36%
	SENS	65.00%	42.10%	47.62%
Selected features by NCA				
K-NN	ACCU	80.00%	55.00%	60.00%
	SPEC	83.33%	54.54%	58.69%
	SENS	78.94%	55.55%	61.76%
SVM	ACCU	85.00%	55.00%	62.50%
	SPEC	81.81%	60.00%	60.46%
	SENS	88.88%	54.17%	62.50%
LDA	ACCU	75.00%	55.00%	56.25%
	SPEC	72.72%	56.25%	58.06%
	SENS	77.77%	55.00%	55.10%

ACCU: accuracy, SPEC: specificity, SENS: sensitivity.

**Fig 3:**
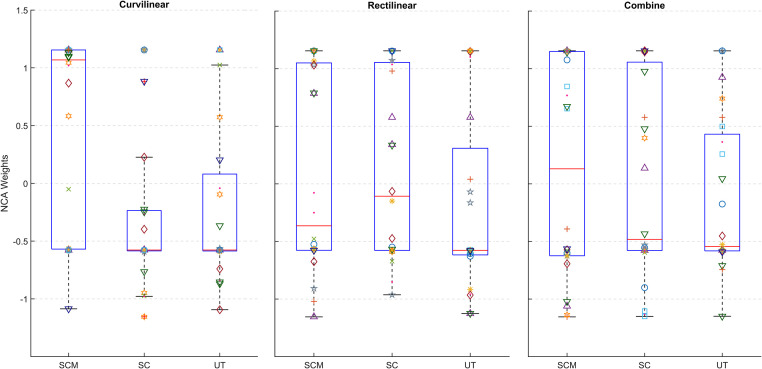
NCA weights of the features for each gait trajectory grouped by muscle. The higher the value, the more important it is. Weights from the left and right muscle are pooled.

Fig 3 shows the analysis carried out by the NCA algorithm. The horizontal coordinate represents the three pairs of neck muscles (SCM, SC and UT) and the vertical coordinate represents the weights of each feature learned by NCA. Each feature is illustrated with a different symbol as shows the bottom legend. This figure allows us to see the distribution of the features for each case. The features which have higher weights are those with more significant value for the classification, and the features with lower weights are irrelevant; the latter were discarded from the dataset. If we compare the weights of each trajectory, we can appreciate that the SCM muscle in curvilinear gait has the highest median (red line). The SCM muscle contains relevant and informative features for CNP identification and therefore leads to better classification accuracy.

**Fig 4:**
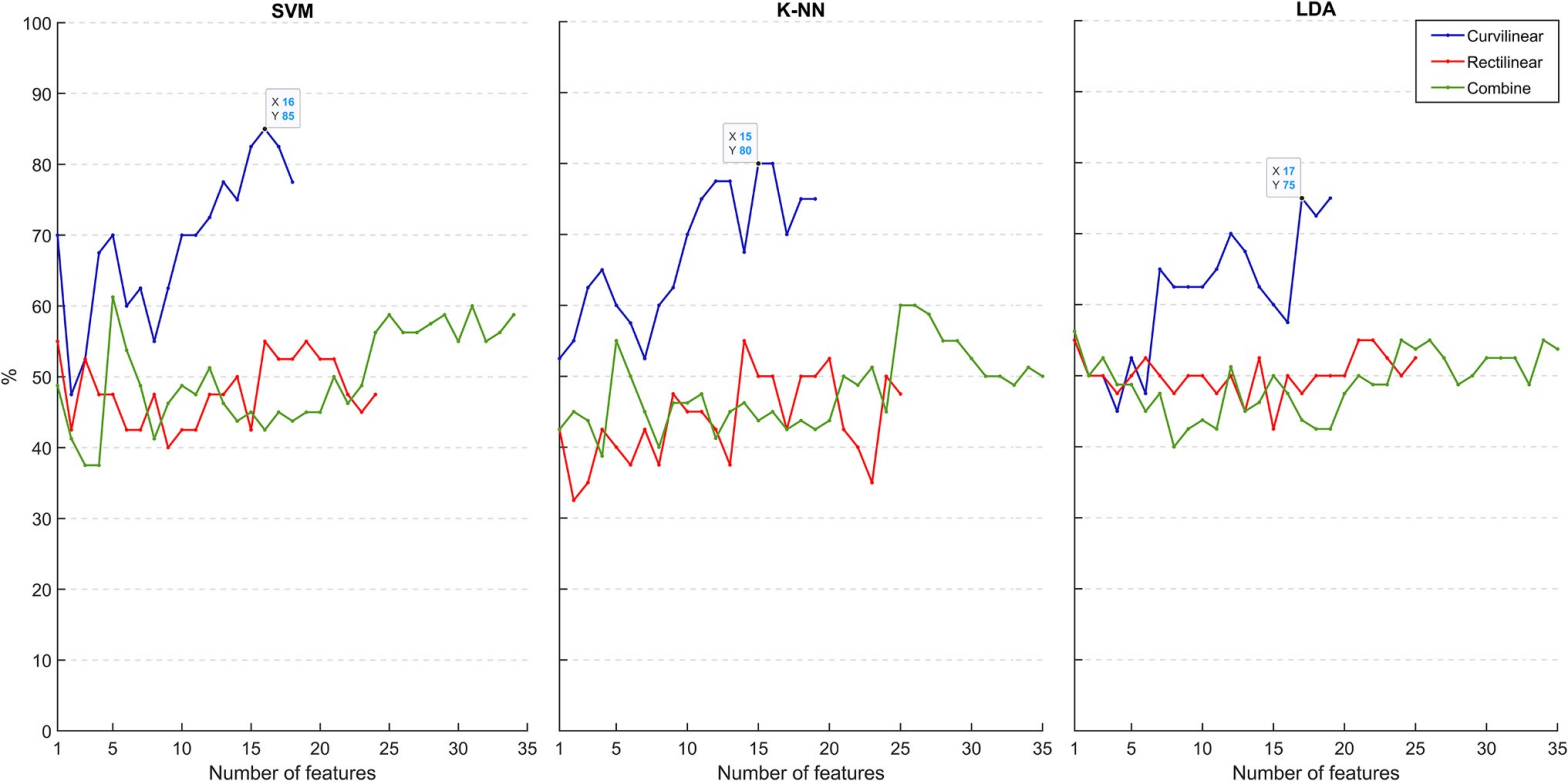
Dependence of % classification accuracy on the number of features selected by NCA.

After the reduction of the number of features based on their weighting factors, we plotted the accuracy as a function of the number of features to find the best classification performance of each algorithm for each trajectory. Fig 4 shows that for gait along the curved path. the SVM classifier achieved 85% with 16 features, the K-NN classifier achieved 80% with 15 features, and LDA achieved 75% with 17 features. Table 4 shows the 16 features that correspond to the highest performance and therefore, those with most discriminative power. Among them, the MSC is a key feature that affects the classification performance, playing a substantial role differentiating between groups. SSI and WL were the other features with the highest power for class separability. Overall, 50% of the features correspond to the time domain and the other half to the frequency domain and more than 50% of the features relate to the SCM muscle.

**Table 4:** Selected features by NCA.

		Curvilinear		Rectilinear		Combine	
Muscles	Side	Features	FW*	Features	FW	Features	FW
SCM	R	VAR MAV SSI MNF PKF	2.409 1.022 0.524 1.168 1.880	MAV	4.097	MAV RMS WL SSI	0.100 2.918 3.902 0.848
	L	VAR SSI PKF MSC	1.817 2.992 1.113 0.852	-	-	VAR	3.565
SC	L	VAR WL MNF PKF	0.881 2.692 1.103 2.616	-	-	-	-
UT	R	MNF MDF	1.046 1.607	-	-	-	-
	L	MAV	0.245	-	-	-	-

*FW: Feature weight (discriminative power), R: right, L: left.

In contrast, features measured during rectilinear gait or combined trajectories lead to poor classification, obtaining a maximum accuracy of 55% and 62.50%, respectively using the SVM algorithm.

**Fig 5:**
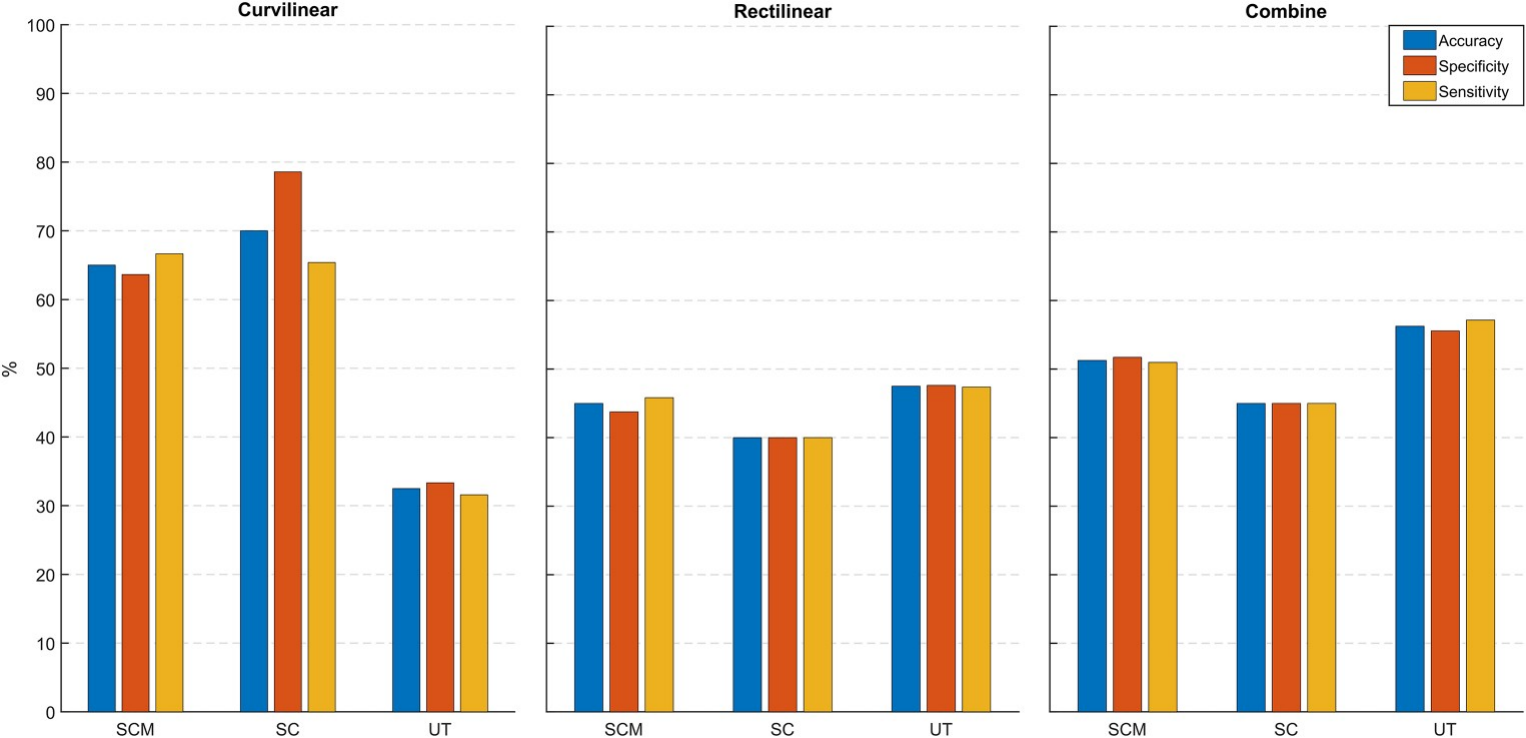
Classification performance for each gait trajectory for each muscle.

Considering the performance of NCA-SVM, we also analyzed the classification power of each muscle individually through this model (see Fig 5). The best classification performance corresponds to the SC muscle during curvilinear gait with an accuracy of 70%, a specificity of 78% and a sensitivity of 60%, followed by SCM with an accuracy of 65%, a specificity of 63.63% and a sensitivity of 66.67%. Compared to the other trajectories, the performance difference between muscles is easily noticeable in curvilinear gait where SCM and SC muscles achieved higher performance than UT muscle and at the same time, higher than the other trajectories.

**Fig 6:**
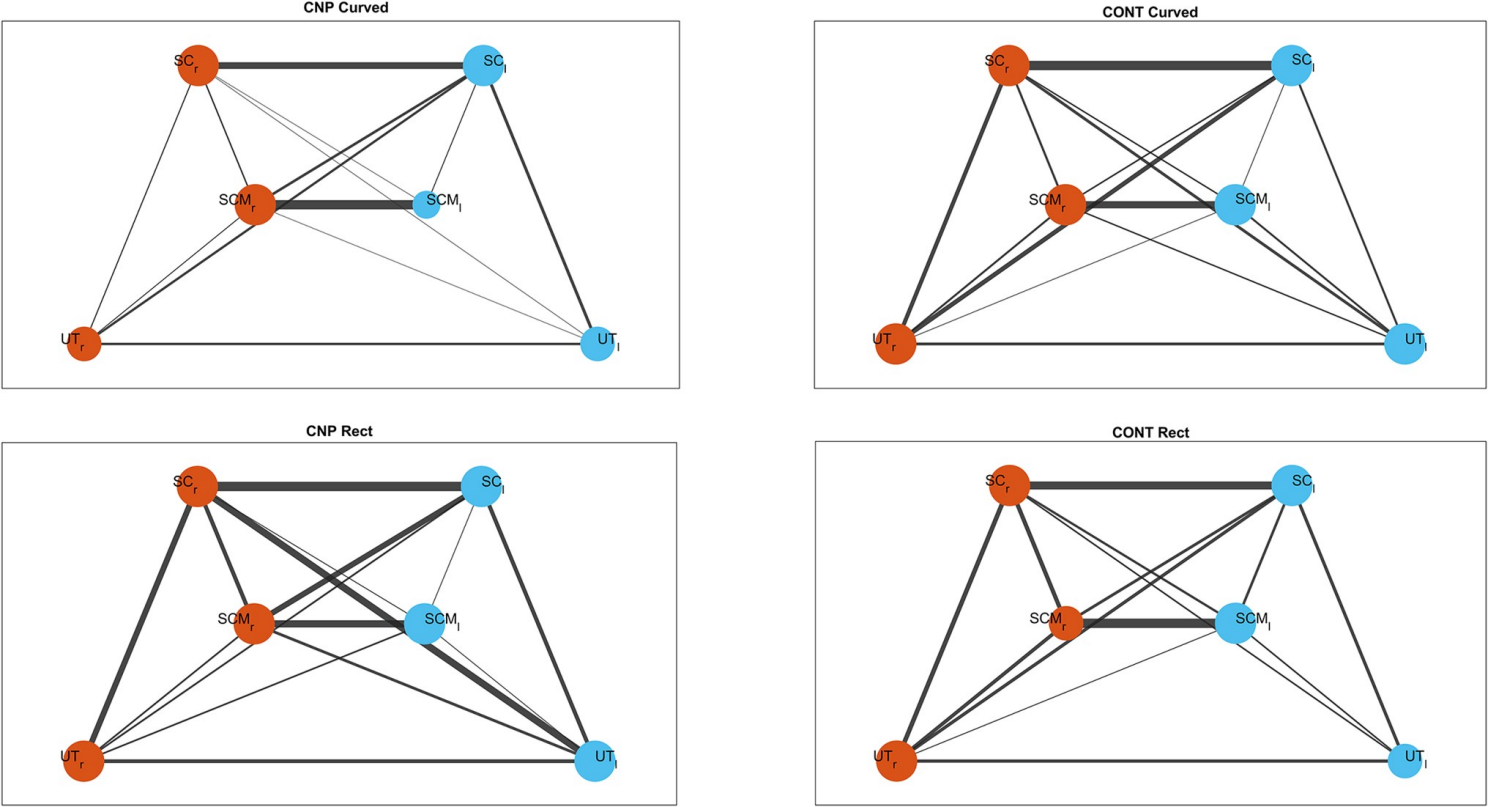
Functional networks of CNP and control groups during curvilinear and rectilinear task at delta band. Orange nodes represents left side muscles and blue nodes the right-side ones.

The topology of complex networks was analyzed to compare functional interactions between neck muscles across different conditions and frequencies for both groups. All network measures were derived from weighted coherence matrices and averaged across nodes for each group, condition, and frequency band. The BC showed significance during curvilinear walking in the delta band (p = 0.021) between those with and without CNP. The BC was not significantly different between groups at the same frequency range (p = 0.068) for rectilinear walking. On the other hand, the strength was not significant between groups (Table 5). No significant differences were found in other frequency bands. Fig 6 shows indirect neck muscle networks in the delta band during curvilinear and rectilinear walking. Node’s size represents the degree, which is the number of edges connected to other nodes (muscles). Connection strength is reflected by the width of the edge. As can be seen in this figure, the muscle network in those with CNP presents thinner edges and a smaller number of connections, which suggests a weaker connectivity network between muscles during curvilinear walking.

**Table 5:** Network parameters.

		Curvilinear	Rectilinear	p
Strength	CNP	0.901±0.06	0.913±0.04	P > 0.05
	Control	0.933±0.04	0.923±0.03	
BC	CNP	0.034±0.02	0.046±0.03	P < 0.05
	Control	0.015±0.06	0.029±0.02	

## Discussion

Several studies have reported altered muscle activity in people with CNP compared to asymptomatic individuals [35, 48]. This study shows that EMG measures of neck muscle activity, especially in SCM and SC, can be used to classify people with and without CNP confirming that differences in neck muscle behavior exist in people with CNP. The best classification performance was achieved during curvilinear gait, suggesting that more simple tasks such as normal rectilinear gait is not sufficient to reveal these differences. In contrast, walking along a nonlinear path requires fine coordination between all body segments as well as anticipatory strategies by the CNS, making it a more complex task [11, 12].

The SVM algorithm with the radial basic function kernel exhibited the best performance with an accuracy of 85.00%, a specificity of 81.81% and a sensitivity of 88.88% during curvilinear gait. Nevertheless K-NN and LDA also performed well during curvilinear gait, achieving an accuracy of 80% and 75%, respectively. Interestingly, for the rectilinear trajectory, the three classifiers obtained the same accuracy of 55% and very similar values in terms of specificity and sensitivity. A similar pattern of results was seen in prior studies where the same classifiers were trained with EMG data from limb muscles. The classifiers showed same performance ranking (SVM with the best classification performance, followed by K-NN and LDA) [49, 50]. Indeed, other studies suggest that K-NN generally performs better than LDA [51].

Overall, since three different algorithms perform better with curvilinear path, we can conclude that nonlinear gait produces more distinguishable features. This could be due to the need for higher muscle activation or coordination between muscles in order to adapt a new strategy for a more complex task, e.g. larger shear forces in the frontal and horizontal planes require more muscle activity to maintain the head centred.

### High impact features and muscle networks

The optimal selection of features for the characterization of EMG signals has been the subject of many studies. Here the NCA, which captures information related to the discrimination power of the features, was used to reduce the number of features. On average, NCA yielded better classification results with a smaller number of features in all supervised models. We can observe in Table 4 that there are more features selected from the SCM muscle, which is consistent with Fig 3 where the SCM in curvilinear gait has a median weight higher than the other muscles. NCA has previously been applied in EMG recognition during gait, showing better performance than other techniques such as PCA and LDA [21].

Features with the highest weights correspond to SSI and WL, which are time-domain features. This supports previous results [25] which report that time-domain features provide better signal classification. Additionally, WL has already been established in some studies as a key feature in EMG identification [41, 52]. Along with these two features, PKF and VAR feature also presented high discriminative value.

Our proposed tool, MSC, was selected by NCA as a key feature and was therefore used for the classification task, achieving high accuracy. This suggests different muscle synergies in people with CNP and the presence of task-specific muscle synergies that appear only when walking along a curved path.

Network topology was carried out to identify the neural synchrony of these muscle synergies. Graphical and statistical differences were found between those with and without CNP during curvilinear gait across the delta band (1-4Hz) [53]. Significant coherence in the delta band has been previously observed during postural tasks and gait due to the strong presence of common synaptic input produced in this band [54]. The delta band is the most relevant band for the generation and control of force. Those with CNP displayed higher values than the control group in the BC, which suggests that in those with CNP, some nodes play a controlling role in the passage of information through the network [44].

### Study limitations

Given the relatively small sample size (20 asymptomatic individuals and 20 individuals with CNP), our results are not easily generalizable to a broader population of people with CNP. Our participants with CNP presented with mild to moderate neck pain intensity and it is also not known whether the same results would be evident in people with more severe CNP or with different neck pain disorders.

Muscle networks are commonly analyzed with a large number of muscles, and therefore considering that our results report on data from a relatively small number of muscles, caution should also be taken when interpreting the results. Nevertheless, this is the first study to apply a network analysis to investigate neck muscle activity and therefore our findings form the important basis of future work.

### Future research

Intermuscular coherence holds promise as a new biomarker for CNP classification not only as a discriminative feature but also as a technique to reveal network topologies. This approach may contribute to the identification of irregular motor coordination associated with the presence of chronic pain. Future research in this field is required with a larger number of patients to explore further task-dependent muscle synergies and how their behavior is modified with pain. These biomarkers may prove useful in longitudinal studies for predicting the transition from acute to chronic pain or recurrent pain episodes.

## Conclusion

This study presents an approach for the classification of CNP based on surface EMG by using intermuscular coherence as a new technique to assess neck muscle coordination. We found different task-specific synergies between asymptomatic people and people with CNP during curvilinear gait, which contributed to the classification accuracy.
